# Platinum versus immunotherapy for unresectable esophageal cancer: A protocol for systematic review and meta analysis

**DOI:** 10.1097/MD.0000000000023537

**Published:** 2020-12-18

**Authors:** Jiekun Qian, Zhangwei Tong, Yannan Zhang, Chun Chen

**Affiliations:** a Department of Thoracic Surgery, Fujian Medical University Union Hospital; b Department of Ophthalmology, The First Affiliated Hospital, Fujian Medical University, Fuzhou, China.

**Keywords:** esophageal cancer, immunotherapy, platinum-based chemotherapy

## Abstract

**Background:**

Esophageal cancer is one of the most common malignant tumors, with early metastasis, highly malignant characteristics. Morbidity ranks 7th among all malignant tumors, and mortality ranks 6th. Esophageal adjuvant therapy can significantly improve overall survival in unresectable esophageal cancer patients. With the breakthrough and progress of immunotherapy, the possibility of curing esophageal cancer has greatly increased. Some clinical trials have reported that compared with traditional platinum-based chemotherapy, the use of programmed death 1 (PD-1) and programmed death ligand 1 (PD-L1) inhibitors alone can benefit patients and effectively prolong their overall survival. We compare the efficacy of single immunotherapy with traditional platinum-based chemotherapy in a systematic review and meta-analysis to provide a reliable basis for clinicians.

**Methods:**

We will search PubMed, Medline, Embase, Web of Science, Cancerlit, Google Scholar, and the Cochrane Central Register of Controlled Trials for related studies published before December 1, 2019 without language restrictions. Two review authors will search and assess relevant studies independently. Randomized controlled trials (RCTs) or quasi-RCTs, and prospective cohort studies will be included. We will perform subgroup analysis in sex, age, ethnicity, and tumor stage of esophageal cancer patients.

**Results:**

The results of this study will be published in a peer-reviewed journal.

**Conclusion:**

The results of this systematic review and meta-analysis will provide a basis for clinicians to formulate the best chemotherapy regimen for patients, as well as a research clue for clinical researchers in this field. The results of this study will expand the treatment options for esophageal patients, but due to the nature of the disease and intervention, large sample clinical trials are not abundant, so we will include some high-quality small sample trials, which may cause high heterogeneity.

**INPLASY registration number:**

INPLASY2020110012.

## 1 Introduction

Esophageal cancer is one of the most common malignancies with a gradual increase in morbidity, ranking 7th in the incidence and 6th in the mortality of all malignancies worldwide.^[1–3]^ Esophageal cancer is a highly malignant tumor with a strong tendency of invasion and metastasis.^[4,5]^ Despite multiple treatment methods, it is still one of the main causes of cancer-related death in the world.^[6]^ The 5-year survival rate of stage I patients was about 90%, while that of stage II patients was reduced to 45%, that of stage III patients was 20%, and that of stage IV patients was only 10%.^[7]^

Patients with esophageal cancer are usually diagnosed in the middle or advanced stages of tumor. The combination of conventional platinum-based chemotherapy and surgical treatment can significantly improve the overall survival rate of patients, but the prognosis of patients with esophageal cancer is still very poor.^[8–11]^ Immunotherapy is a relatively new field in the treatment of esophageal cancer. Some clinical trials reported that programmed death 1 (PD-1) and programmed death ligand 1 (PD-L1) inhibitors alone have better application prospects than platinum-based chemotherapy.^[12–18]^ We will conducted a systematic review and meta-analysis on the efficacy comparison between immunotherapy and traditional platinum-based chemotherapy, so as to provide a reliable basis for further promotion of immunotherapy and for clinicians to formulate the best chemotherapy regimen for patients with unresectable esophageal cancer.

## 2 Objective

We will evaluate the efficacy of postoperative adjuvant therapy (platinum based chemotherapy and immunotherapy) with or without radiotherapy for patients with unresectable esophageal cancer.

## 3 Methods

This protocol is conducted according to the Preferred Reporting Items for Systematic Review and Meta-Analysis Protocols (PRISMA-P) statement.^[19]^ We will report the results of this systematic review and meta-analysis adhere to the Preferred Reporting Items for Systematic Reviews and Meta-Analyse (PRISMA) guidelines.^[19]^ This protocol has been registered in the INPLASY network (registration number: INPLASY2020110012).

### 3.1 Patient and public involvement

This study will be based on published or unpublished studies and records and will not involve patients or the public directly.

### 3.2 Eligibility criteria

#### 3.2.1 Types of studies

Randomized controlled trials (RCTs) and quasi-RCTs published or unpublished will be included, which have been completed and compared postoperative platinum-base chemotherapy versus immunotherapy for patients with unresectable esophageal cancer.

#### 3.2.2 Types of participants

The participants will be adults diagnosed with unresectable esophageal cancer histologically or cytologically confirmed who were treated with platinum-based chemotherapy, or immunotherapy. No restrictions on ethnicity, sex, education, and economic status will be applied.

#### 3.2.3 Types of interventions

According to the means of postoperative chemotherapy for patients with unresectable esophageal cancer, the trials included will be divided into the following categories.

- Immunotherapy versus molecular targeted therapy.
- Immunotherapy versus anti-angiogenic agents.
- Postoperative platinum-base chemotherapy versus molecular targeted therapy.
- Platinum-based chemotherapy versus anti-angiogenic agents.
- Platinum-based chemotherapy versus immunotherapy.

#### 3.2.4 Types of outcome measures

##### 3.2.4.1 Primary outcomes

The primary outcomes will be postoperative overall survival of patients with unresectable esophageal cancer who were treated with chemotherapy.

#### 3.2.5 Secondary outcomes

We will assess the 5-year survival, median survival, recurrence-free survival, quality of life, and adverse events or complications of patients with unresectable esophageal cancer who were treated with chemotherapy.

### 3.3 Information sources

We will search PubMed (Medline), Embase, Google Scholar, Cancerlit, and the Cochrane Central Register of Controlled Trials for related studies published before June 20, 2021 without language restrictions.

### 3.4 Search strategy

We will use the relevant keywords or subject terms adhered to Medical Subject Heading (MeSH) terms to search for eligible studies in the electronic databases which were mentioned above without language restrictions. The PubMed search strategies are shown in Table 1.

**Table 1:** PubMed search strategies.

Query	Search term
# 1	Esophageal Neoplasm OR Neoplasm, Esophageal OR Esophagus Neoplasm OR Esophagus Neoplasms OR Neoplasm, Esophagus OR Neoplasms, Esophagus OR Neoplasms, Esophageal OR Cancer of Esophagus OR Cancer of the Esophagus OR Esophagus Cancer OR Cancer, Esophagus OR Cancers, Esophagus OR Esophagus Cancers OR Esophageal Cancer OR Cancer, Esophageal OR Cancers, Esophageal OR Esophageal Cancers
# 2	Platinum-based chemotherapy OR Chemotherapy OR Chemotherapies OR Docetaxel OR Taxotere OR Docetaxel OR Pemetrexed OR Alimta OR Pemetrexed OR Cisplatin OR Carboplatin
# 3	Immunotherapy OR Immunotherapies OR Immunosuppression OR PD1 inhibitors OR PDL1 inhibitors
# 4	Randomized controlled trial OR Controlled clinical trial OR Randomized OR Placebo OR Drug therapy OR Randomly OR Trial OR Groups NOT Animals
# 5	# 1 AND # 2 AND # 3 AND # 4

### 3.5 Data collection and analysis

We will utilize the measures described in the Cochrane Handbook for Systematic Reviews of Interventions to pool the evidence.^[20]^

#### 3.5.1 Study selection

Two reviewers (JKQ, ZWT) will investigate each title and abstract of all literatures searched independently and identify whether the trials meet the inclusion criteria as designed and described in this protocol. Two authors (JKQ, ZWT) will in duplicate and independently screen the full text of all potential eligible studies to exclude irrelevant studies or determine eligibility. The 2 reviewers will list all the studies included and document the primary reasons of exclusion for studies that do not conform to the inclusion criteria. Disagreements between the 2 authors will be resolved by discussing with the third author (YNZ), if necessary, consulting with the fourth author (CC). We will show the selection process in details in the PRISMA flow chart.

#### 3.5.2 Data extraction and management

The 2 authors (JKQ, ZWT) will extract the following data independently from the studies included.

- Study characteristics and methodology: publication date, the first author, country, randomization, study design, periods of data collection, follow-up duration, total duration of study, and withdrawals, etc
- Participant characteristics: sex, age, tumor stage, pathology diagnosis, ethnicity, performance status, history of smoking, pathologic tumor size, and inclusion criteria, etc.
- Interventions: therapeutic means, drugs, dosage, modality and frequency of administration, etc.
- Outcome and other data: overall survival, 5-year survival, median survival, disease-free survival, 95% confidence intervals, recurrence time, quality of life, adverse events, and complications, etc.

We will record all the date extracted in a predesigned table and consult the first author of the trial by e-mail before determining eligibility, if the reported data of which are unclear or missing.

### 3.6 Assessment of risk of bias in included studies

Two authors (JKQ, ZWT) will use the Cochrane Handbook for Systematic Reviews of Interventions to assess the risk of bias of each study included independently based on the following ranges: random sequence generation (selection bias); allocation concealment (selection bias); blinding of participants and personnel (performance bias); blinding of outcome assessment (detection bias); incomplete outcome data (attrition bias); selective outcome reporting (reporting bias); other bias.^[21]^ Each domain will be assessed as high, low, or uncertain risk of bias. The results and details of assessment will be reported on the risk of bias graph.

### 3.7 Data analysis

The data will be synthesized by Review Manager 5.3 software. We will conduct a systematic review and meta-analysis only if the data gathered from included trials are judged to be similar enough to ensure a result that is meaningful. The chi-sqared test and *I*^2^ statistic will be used to assess statistical heterogeneity among the trials included in matched pairs comparison for standard meta-analysis. The random effect model will be applied to analyze the data, if there is substantial heterogeneity (*P* < .1 or *I*^2^ statistic >50%) and the trials will be regarded to be obvious heterogeneous. Otherwise, we will utilize fixed effect model to analyze the data. Mantel–Haenszel method will be adopted to pool of the binary data. The results will be reported in the form of relative risk (RR) between 95% confidence interval (CI) of the date. The continuous data will be pooled by inverse variance analysis method and the results will be shown in the form of standardized mean difference (SMD) with 95% confidence interval (CI) of the date.

#### 3.7.1 Subgroup analysis

If there is high heterogeneity (*I*^2^ statistic >50%) and the data are sufficient, subgroup analysis will be conducted to search potential causes of heterogeneity. Subgroup analysis will be performed in different methods of postoperative adjuvant therapy, ethnicity, history of smoking, tumor stage, and type of operation.

#### 3.7.2 Sensitivity analysis

Sensitivity analysis will be conducted to assess the reliability and robustness of the aggregation results via eliminating trials with high bias risk.

### 3.8 Publication bias

If there are 10 or >10 trials included, we will construct a funnel plot and use Egger test to assess publication bias. If reporting bias is suspected, we will consult the study author to get more information. If publication bias does exist, we will apply the fill and trim method to analyze publication bias in the trials.^[22]^

### 3.9 Evidence evaluation

We will evaluate all the evidence according to the criteria of GRADE (imprecision, study limitations, publication bias, consistency of effect, and indirectness bias). The quality of all evidence will be evaluated as 4 levels (high, moderate, low, and very low).^[23]^

## 4 Discussion

Esophageal cancer is a highly malignant tumor. Although there are many advanced treatment methods combined with surgical treatment, the prognosis of patients is very poor. Esophagectomy is the main treatment for early esophageal cancer, but the esophageal cancer that is often detected is already advanced. Adjuvant therapy plays a key role, which is a key factor that contributes to the overall survival of patients. Esophageal cancer mainly occurs in middle-aged and elderly patients, whose quality of life and physical fitness are poor. Therefore, what we need to pursue now is therapies that can significantly improve overall survival rates with fewer side effects. Immunotherapy is a new field in the treatment of esophageal cancer. Many trials have reported that programmed death 1 and programmed death ligand 1 inhibitors can benefit patients more than traditional platinum-based chemotherapy. We will conduct a systematic, comprehensive and objective assessment of immunotherapy and platinum-based adjuvant chemotherapy. The results of this study will provide the basis for clinicians to formulate the best postoperative adjuvant treatment strategies for patients with esophageal cancer, and provide scientific clues for researchers in this field.

## Author contributions

**Conceptualization:** Jiekun Qian, Zhangwei Tong, Yannan Zhang, Chun Chen.

**Data curation:** Jiekun Qian, Zhangwei Tong, Chun Chen.

**Formal analysis:** Jiekun Qian, Zhangwei Tong, Yannan Zhang, Chun Chen.

**Funding acquisition:** Yannan Zhang, Chun Chen.

**Investigation:** Jiekun Qian, Chun Chen.

**Methodology:** Jiekun Qian.

**Project administration:** Jiekun Qian.

**Resources:** Jiekun Qian.

**Software:** Jiekun Qian.

**Supervision:** Chun Chen.

**Validation:** Jiekun Qian.

**Writing – original draft:** Jiekun Qian, Chun Chen.

**Writing – review & editing:** Jiekun Qian, Chun Chen.
